# What is the best first-line treatment for POEMS syndrome: autologous transplantation, melphalan and dexamethasone, or lenalidomide and dexamethasone?

**DOI:** 10.1038/s41375-019-0391-2

**Published:** 2019-01-30

**Authors:** Hao Zhao, Xu-fei Huang, Xue-min Gao, Hao Cai, Lu Zhang, Jun Feng, Xin-xin Cao, Dao-bin Zhou, Jian Li

**Affiliations:** Department of Hematology, Peking Union Medical College Hospital, Chinese Academy of Medical Sciences and Peking Union Medical College, Beijing, China

**Keywords:** Combination drug therapy, Myeloma

## Abstract

POEMS syndrome is a rare plasma cell dyscrasia. This study compared the responses to and survival of 347 POEMS syndrome patients given three first-line treatment regimens: autologous stem cell transplantation (ASCT, *N* = 165) and melphalan + dexamethasone (MDex, *N* = 79), or lenalidomide + dexamethasone (LDex, *N* = 103). After a median 45-month follow-up, overall hematologic complete remission (CR_*H*_) was 46.4%, vascular endothelial growth factor complete remission (CR_*V*_) was 55.1%, and neurological remission (R_*N*_) was 93.8%. CR_*H*_ was better with ASCT (49.7%) than with MDex (37.7%, *p* = 0.001). CR_*V*_ was better with ASCT (66.2%) than with MDex (38.5%, *p* = 0.001) or LDex (47.7%, *p* = 0.008). Differences in R_*N*_ achieved by three regimens (91.5% vs. 100% vs. 93.8%, *p* = 0.234) were not significant. Overall 3-year progression-free survival (PFS) was 80.5% and overall 3-year overall survival (OS) was 90.8%. PFS was 87.6% with ASCT and 64.9% with LDex (*p* = 0.003). OS in the three regimens did not differ (*p* = 0.079). In medium-high risk patients, ASCT had better CR_*H*_ and CR_*V*_ than MDex, and better PFS than LDex. Therefore, although all three treatments had reasonable responses and survivals, patients with higher risk may benefit more from ASCT treatment.

## Introduction

POEMS syndrome is a rare plasma cell dyscrasia characterized by polyneuropathy, organomegaly, endocrinopathy, monoclonal gammopathy, and skin changes. There is currently no standard treatment [1]. Current systematic therapies include high-dose chemotherapy with autologous stem cell transplantation (ASCT), alkylator-based therapy, and therapy with novel agents. High-dose chemotherapy with ASCT can achieve excellent clinical responses, with a significant improvement of neuropathy, complete hematologic response (CR_*H*_) in 60% of POEMS syndrome patients, and complete VEGF response (CR_*V*_) in 50%. Five-year progression-free survival (PFS) of 75% and 5-year overall survival (OS) of 90% have been reported [2–4]. However, treatment-related complications are frequent, with peri-engraftment syndrome reported in 12–50% of patients [5, 6]. Melphalan is an alkylation agent that is widely used for treatment of plasma disorders including myeloma and amyloidosis [7]. Melphalan plus dexamethasone (MDex) achieved 100% neurological response (R_*N*_), 38.7% CR_*H*_, and 95.8% CR_*V*_ in a prospective study of 31 POEMS syndrome patients, but long-term outcomes were not reported [8, 9]. MDex is less toxic than ASCT, with no treatment-related mortality and a low incidence of severe adverse events. Lenalidomide is an immunomodulatory drug that is also cytotoxic to malignant plasma cells [10]. Lenalidomide plus dexamethasone (LDex) achieved an R_*N*_ of over 95%, a CR_*H*_ of 46.3%, a CR_*V*_ of 42.5%, a 3-year PFS of 75%, and an OS of 90% in a phase II single-arm prospective study including 41 patients [11]. All three treatments can achieve acceptable remission rates and survival, but it is unclear which is the best first-line treatment for POEMS syndrome. The efficacy of these treatments has not previously been compared in large patient cohorts, but the rarity of the disease makes it difficult to conduct randomized controlled trials. This study retrospectively evaluated the treatment efficacy and survival of the three treatment regimens in cohort of newly diagnosed POEMS syndrome patients treated at a single center.

## Patients and methods

### Patients

The medical records of POEMS syndrome patients admitted to our hospital between January 2000 and October 2017 and initially treated with ASCT, MDex, or LDex were evaluated. A total of 347 patients who satisfied the POEMS syndrome diagnosis criteria described by Dispenzieri et al. and had complete treatment and follow-up data were included [1]. All patients provided informed consent. The study was approved by the Institutional Review Board of Peking Union Medical College Hospital and conducted following the ethical guidelines of the Declaration of Helsinki.

### Risk stratification

Patients were stratified to low, medium, or high risk groups using four baseline characteristics, age > 50 years, presence of pulmonary hypertension, presence of pleural effusion, and an estimated glomerular filtration rate < 30 ml/min/1.73 m^2^. The first three characteristics had a value of 1; the last had a value of 2. Patients with total scores 0, 1, and 2–5 were assigned to low, medium, and high risk groups, respectively [12].

### Treatment regimens

As no standard algorithm is available to guide the choice of treatment, the choice of regimen was mainly based on the clinician’s judgment. Patients eligible for ASCT were < 65 years of age, without serious systemic disease or organ dysfunction, severe pulmonary hypertension, severe capillary leak syndrome (hypotension and/or refractory ascites), and active infection, and with successful collection of adequate peripheral blood stem cells [13]. The protocol included mobilization with cyclophosphamide 3 g/m^2^ and colony-stimulating factor and conditioning with high-dose melphalan 140–200 mg/m^2^. MDex therapy included melphalan 10 mg/m^2^ + dexamethasone 40 mg on days 1–4, one cycle every 28 days for a total of 9 cycles. LDex therapy included lenalidomide 10–25 mg on days 1–21, dexamethasone 40 mg on days 1, 8, 15, 22, one cycle every 28 days for a total of 12 cycles. Prophylaxis of embolization with LDex therapy was by aspirin 100 mg qd.

### Response criteria

CR_*H*_ was assessed by immunofixation electrophoresis and free immunoglobulin light-chain negativity in both serum and urine. CR_*V*_ was defined as a decrease in VEGF to a normal concentration (<600 pg/ml). Achieving either CR_*H*_ or CR_*V*_ was defined as CR_*H*_/CR_*V*_. Neurological dysfunction was one of the key clinical manifestations and affects patients’ life quality the most. Therefore, we use R_*N*_ to represent clinical remissions. R_*N*_ was defined as a decrease in the Overall Neuropathy Limitations Scale (ONLS) score of at least 1 point [8].

### Statistical analysis

PFS was the time from treatment to recurrence, the deterioration of clinical symptoms, or death. OS was defined as the time from treatment to death. The date of last follow-up was August 31, 2018. Statistical analysis was performed with SPSS 22 (SPSS, Inc., Chicago, IL, USA). The chi-square test, or Fisher's exact test when appropriate, was used to determine the significance of differences in the values of categorical variables. Time to responses were calculated from the date of treatment initiation. Survival curves were plotted by the Kaplan–Meier method and differences were compared with the log-rank test. *p*-Values < 0.05 were considered significant.

## Results

### Patient characteristics

The patient baseline characteristics are shown in Table 1. The median age at the time of diagnosis was 48 (range, 21–74) years; 65.7% were men. The median ONLS score was 4 of a possible 0–12 points, and peripheral neuropathy was confirmed by electromyography in the 19 patients with ONLS scores of 0. A total of 61.1% of the patients were positive for IgA type heavy chain monoclonal immunoglobulin. The light chains were all λ type. Risk stratification resulted in 118 low-risk (34.0%), 150 medium-risk (43.2%), and 79 high-risk (22.8%) patients.

**Table. 1:** Baseline characteristics of POEMS syndrome patients

Baseline charaterisitics	All patients(*N* = 347)	MDex(*N* = 79)	ASCT(*N* = 165)	LDex(*N* = 103)	*p-*value
Age > 50 years	40.3	48.1	32.7	46.6	0.022
Male	65.7	67.1	68.5	60.2	0.364
Polyneuropathy ONLS > 4	38.9	46.8	38.8	33.0	0.166
*Organomegaly*					
Hepatomegaly	38.3	43.0	40.6	31.1	0.238
Splenomegaly	59.1	57.0	67.9	47.6	0.008
Lymphadenopathy	64.0	69.6	60.6	65.0	0.376
IgA type heavy chain	61.1	69.6	54.5	65.0	0.048
SPE > 5 g/l	10.7	8.9	11.5	10.7	0.821
BMPC > 10%	3.7	1.3	4.8	3.9	0.441
Angioma	59.4	55.7	52.1	68.9	0.062
Peripheral edema	84.1	91.1	74.5	94.2	0.000
Ascites	43.2	55.7	32.7	50.5	0.001
Pleural effusion	37.8	50.6	29.1	41.7	0.003
Castleman disease^a^	61.5(*N* = 52)	41.7(*N* = 12)	65.5(*N* = 29)	2.7(*N* = 11)	0.249
Papilledema	66.0	72.2	66.1	60.2	0.330
Osteosclerosis	81.6	68.4	86.1	81.6	0.056
Hypoalbuminemia (< 30 g/l)	13.0	19.0	10.3	12.6	0.167
Renal dysfunction (eGFR < 30 ml/min/ 1.73 m^2^)	4.6	5.1	3.6	5.8	0.691
24 h urinary protein > 1 g	5.8	5.1	4.2	7.8	0.482
VEGF > 4000 pg/ml	57.6	41.8	58.8	67.0	0.004
*Risk stratification*					
Low risk	34.0	22.8	44.2	26.2	0.001
Medium risk	43.2	43.0	43.0	42.7	0.994
High risk	22.8	34.2	12.7	30.1	0.000

The numbers were all presented as percentage

*ASCT* autologous stem cell transplantation, *Alb* albumin, *eGFR* estimated glomerular filtration rate, *IgG* immunoglobulin G, *ONLS* Overall Neuropathy Limitations Scale, *SPE* serum protein electrophoresis, *VEGF* vascular endothelial growth factor

^a^Castleman’s disease was diagnosed in 32 of 52 patients (61.5%) who underwent tissue biopsies

Of 347 included patients, 79 received MDex, 103 received LDex, and 165 were treated by ASCT. Compared with the MDex or LDex groups, patients treated with ASCT had a more favorable baseline clinical manifestations, with a lower percentage of patients > 50 years of age (*p* = 0.022), an extravascular water load (*p* = 0.001), or IgA type heavy chains (*p* = 0.048). Fewer high-risk patients were treated with ASCT than with MDex or LDex (*p* < 0.001).

### Treatment response

Overall median follow-up was 45 months, total follow-up time was 1420 person-years. Median follow up was 90 months for MDex patients, 48 months for ASCT patients, and 44 months for LDex patients.

**Fig. 1:**
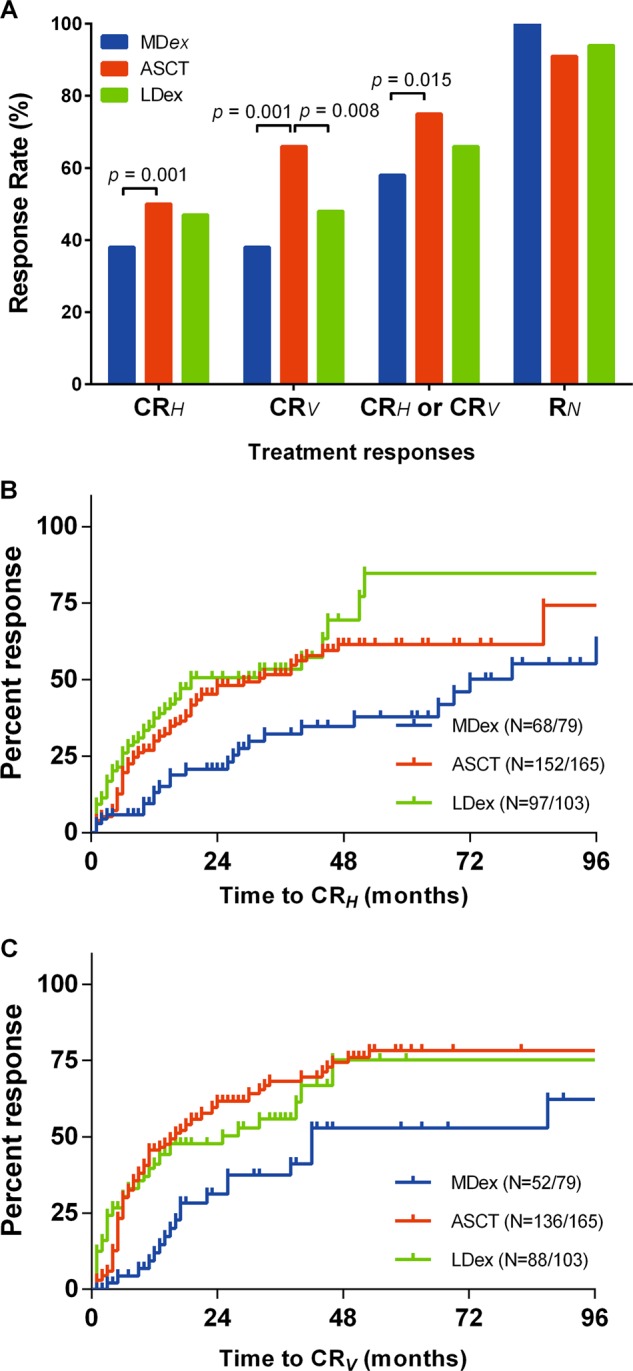
Therapeutic efficacy and time to responses of ASCT, MDex, and LDex. **a** Hematologic complete remission, VEGF complete remission, and neurological remission rates. **b** Time to response for CR_*H*_. **c** Time to response for CR_*V*_

The total CR_*H*_ rate was 46.4%. It was higher with ASCT (49.7%) than with MDex (37.7%, *p* = 0.001). Subgroup analysis found that the regimen-associated difference was mainly in medium-high risk patients (57.0% vs. 35.8%, *p* = 0.023), and the CR_*H*_ responses in the low-risk patients (40.6% vs. 43.8%, *p* = 1.000) were not significantly different. The CR_*H*_ rates in the LDex (49.7%) and ASCT (47.5%) groups were not significantly different (*p* = 0.797). (Fig. 1a).

The total CR_*V*_ rate was 55.1%. CR_*V*_ was significantly better after ASCT than after MDex (38.5%, *p* = 0.001) or LDex (47.7%, *p* = 0.008). (Fig. 1a) Subgroup analysis found that the CR_*V*_ benefit of ASCT compared with the other regimens was significant in medium-high risk (*p* = 0.001), but not in low-risk (*p* = 0.222). Moreover, CR_*V*_ was prognostic for PFS in medium-high risk patients, but not for OS in these groups. (Supplement Fig. 1).

Combined CR_*H*_ and CR_*V*_ have been associated with PFS and OS, and can be used as a surrogate endpoint [14]. The overall CR_*H*_/CR_*V*_ rate was 65.8% and the ASCT rate (75.3%) was higher than that with MDex (58.3%, *p* = 0.015) but not significantly different from the rate with LDex (*p* = 0.142). The difference in the CR_*H*_/CR_*V*_ rates in the ASCT and MDex groups was observed in medium-high risk patients (*p* = 0.001), but not in low-risk patients (*p* = 0.748).

As for time to response, the overall median time to CR_*H*_ was 10 months (1–179 months). The median time to CR_*H*_ was 26.5 months (1–179 months) for MDex, 8.5 months (1–86 months) for ASCT and 7 months (1–46 months) for LDex, respectively. The total 6-months and 12-months CR_*H*_ rates were 19.2% and 26.1%. The 6-months CR_*H*_ rate was 13.8% for ASCT, 5.9% for MDex and 21.4% for LDex. The 12-months CR_*H*_ rate was 27.9% for ASCT, 9.5% for MDex and 37.4% for LDex (Fig. 1b). The overall median time to CR_*V*_ was 8 months (1–163 months). The median time to CR_*V*_ was 17 months (1–163 months) for MDex, 8 months (1–140 months) for ASCT, and 4 months (1–46 months) for LDex, respectively. The total 6-months and 12-months CR_*V*_ rates were 21.4% and 38.3%. The 6-months CR_*V*_ rate was 23.8% for ASCT, 7.2% for MDex, and 29.7% for LDex. The 12-months CR_*V*_ rate was 46.1% for ASCT, 16.1% for MDex and 37.7% for LDex (Fig. 1c).

The differences in R_*N*_ in the three treatment regimens, 100% with MDex, 91.5% with ASCT, and 93.8% with LDex were not significant (*p* = 0.234) (Table 2).

**Table. 2:** Overall and risk subgroup analysis of best responses and survivals in three treatments

Treatment	All(*N* = 387)		Low risk(*N* = 118)		Medium-high risk (*N* = 229)	
	Rate	*p*	Rate	*p*	Rate	*p*
CR_*H*_						
ASCT	49.7		40.6		57.0	
MDex	37.7	0.001	43.8	1.000	35.8	0.023
LDex	47.5	0.797	48.0	0.638	47.3	0.267
CR_*V*_						
ASCT	66.2		62.1		70.0	
MDex	38.5	0.001	58.3	0.804	32.5	< 0.001
LDex	47.7	0.008	41.7	0.097	50.0	0.022
CR_*H*_/CR_*V*_						
ASCT	75.3		70.0		80.0	
MDex	58.3	0.015	76.9	0.748	53.2	0.001
LDex	66.3	0.142	56.0	0.204	70.1	0.167
R_*N*_						
ASCT	91.5		91.4		91.5	
MDex	100.0	0.188	100.0	1.000	100.0	0.295
LDex	93.8	0.755	100.0	0.543	91.8	1.000
	3y-PFS	*p*	3y-PFS	*p*	3y-PFS	*p*
PFS						
ASCT	87.6		83.7	0.612	84.6	
MDex	84.6	0.568	87.8	0.351	84.6	0.364
LDex	64.9	0.003	59.0	0.016	67.7	0.037
	3y-OS	*p*	3y-OS	*p*	3y-OS	*p*
OS						
ASCT	94.4		96.9		92.3	
MDex	90.7	0.088	94.3	0.701	89.8	0.155
LDex	83.1	0.067	95.8	0.826	78.6	0.099

### Survival

**Fig. 2:**
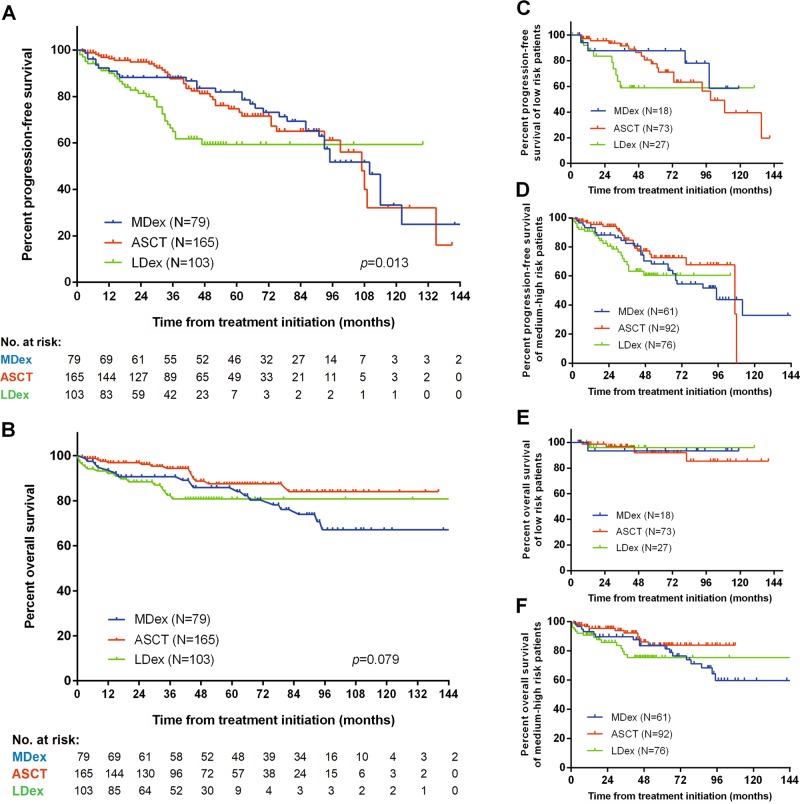
Kaplan–Meier curves of OS and PFS of all, low-risk, medium-risk, and high-risk patients treated with ASCT, MDex, or LDex (**a**) PFS in all patients, (**b**) OS in all patients, (**c**) PFS in low-risk patients, (**d**) PFS in medium-high risk patients, (**e**) OS in low-risk patients, and (**f**) OS in medium-high risk patients

Forty-nine of the 347 patients died during follow-up; two from lethal complications during ASCT. Fifty-one patients experienced disease progression and were alive at the end of the follow-up period. The overall 3-year PFS rate was 80.5% and was significantly higher in ASCT (87.6%) than in LDex (64.9%) patients (*p* = 0.003) but was not different from patients in the MDex group (84.6%, *p* = 0.568) (Fig. 2a). Subgroup analysis showed that PFS was longer with ASCT than with LDex in both low-risk (*p* = 0.016) and medium-high risk patients (*p* = 0.037) (Fig. 2c, d). Overall 3-year OS was 90.4% and did not differ among the treatment groups (94.4% vs. 90.7% vs. 83.1%, *p* = 0.079) (Fig. 2b).

### Risk of secondary malignancy

One secondary malignancy (hepatocellular carcinoma) was reported in the ASCT group. No secondary malignancies were reported in the LDex or MDex groups.

## Discussion

There are currently no standard treatments for patients with POEMS syndrome. ASCT, LDex, and MDex are all effective, but the question “what is the best treatment for POEMS syndrome?” has not been answered [12]. The rarity of POEMS syndrome makes it difficult to conduct randomized double-blind-controlled trials. Consequently, most have been retrospective analyses of single regimens or single-arm prospective studies. This retrospective analysis of 347 patients compared the three most common treatment options in POEMS syndrome, each with a different therapeutic basis. In this patient series, ASCT had the highest response and PFS rates of the three regimens, especially for patients at medium-to-high risk. One explanation is that ASCT eradicates the underlying plasma cell clones more completely than LDex or MDex. However, there were fewer high-risk patients and more patients with mild clinical manifestations in the ASCT group than in the MDex and LDex groups, which included patients with severe organ dysfunction. OS was not significantly better in ASCT patients than in the other groups, possibly because of treatment-related mortality (1.2%), and long-term complications, such as secondary malignancy (0.6%). On the other hand, OS can be improved with second-line treatments for patients with suboptimal responses. Good responses have been achieved with a wide range of treatment choices for refractory POEMS syndrome patients, which partly compensates for deficits in maintaining long-term PFS with first-line treatment. Salvage treatment with lenalidomide or bortezomib-based regimens have both shown promising response rates [15–17]. Novel regimens including daratumumab may further broaden the treatment options for multidrug-resistant patients [18].

MDex and LDex regimens have both been shown effective in POEMS syndrome patients not eligible for ASCT, but it is not clear that they are less toxic alternatives to ASCT as first-line POEMS therapy [8, 19]. In these patients, MDex regimens achieved response rates similar to ASCT in low-risk patients, but response was generally inferior in medium-high risk patients. However, long-term outcomes with MDex and ASCT were not significantly different. Melphalan therapy was generally not favored because of concerns of treatment-related stem cell damage, secondary myelodysplastic syndrome, and leukemia in myeloma patients, with a cumulative risk of 10–15% [20, 21]. However, secondary malignancies or myelodysplastic syndrome were not reported in this patient series. The 3.2% occurrence of secondary malignancies reported in a Japanese cohort of POEMS patients was much lower than that seen in myeloma [2].

Lenalidomide was highly active in these newly diagnosed POEMS syndrome patients, with response rates and OS similar to those with ASCT. However, CR_*H*_ and CR_*H*_/CR_*V*_ or after LDex treatment were not translated into long-term PFS. PFS after LDex treatment was inferior to that achieved with ASCT, even in low-risk patients. This may result from lack of maintenance treatment. Therefore, maintenance therapy was suggested for LDex group because limited cycles of LDex seemed insufficient to maintain long-term PFS compared with ASCT, though it remains to be shown whether adding maintenance treatment can increase PFS as did in myeloma patients [22].

In conclusion, all three treatments had reasonable responses and survivals in newly diagnosed POEMS syndrome patients. However, ASCT might have better responses and survivals compared with the other two groups, especially for higher risk patients. The treatment should be based on a comprehensive evaluation of the physical and the economic status of the patient, the planned follow-up, and the experience of the physicians and treatment center. ASCT could be recommended for high-risk patients if they are eligible. Lenalidomide maintenance after LDex treatment may be beneficial in term of PFS.

## Supplementary information

Supplement Figure 1
