# Supplementary figures and images for: What is the best first-line treatment for POEMS syndrome: autologous transplantation, melphalan and dexamethasone, or lenalidomide and dexamethasone?
Source: Leukemia. 2019 Jan 30;33(4):1023–9. doi: 10.1038/s41375-019-0391-2 (PMC6756085; doi:10.1038/s41375-019-0391-2)

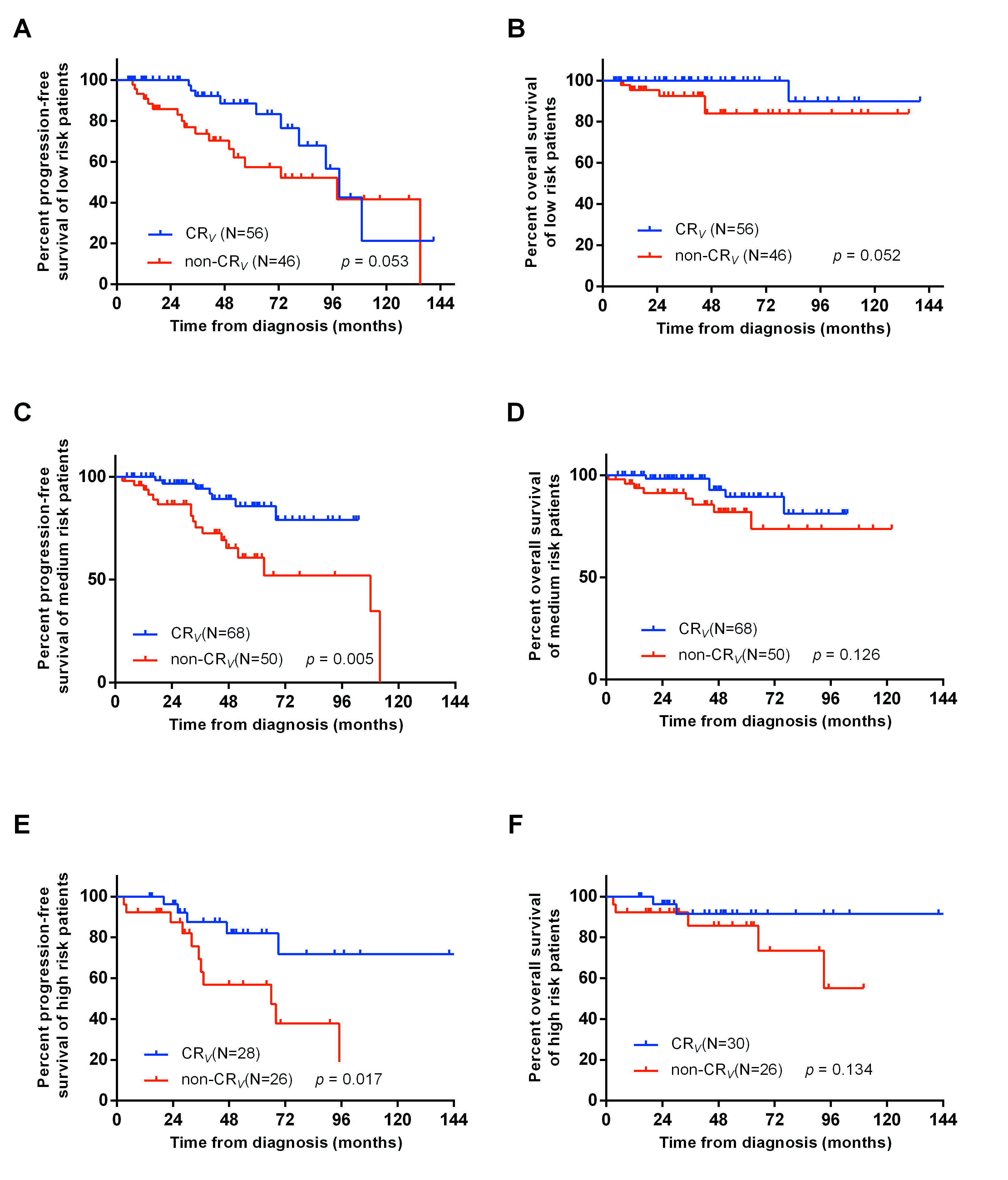

Supplement: Supplementary file 1 — Supplement Figure 1 [file 41375_2019_391_MOESM1_ESM.tif]
